# GOx-assisted synthesis of pillar[5]arene based supramolecular polymeric nanoparticles for targeted/synergistic chemo-chemodynamic cancer therapy

**DOI:** 10.1186/s12951-021-01237-0

**Published:** 2022-01-11

**Authors:** Jin Wang, Di Wang, Moupan Cen, Danni Jing, Jiali Bei, Youyou Huang, Jiannan Zhang, Bing Lu, Yang Wang, Yong Yao

**Affiliations:** School of Chemistry and Chemical Engineering, Nantong University, Nantong, Jiangsu 22 6019 People’s Republic of China

**Keywords:** Pillar[5]arene, Supramolecular polymers, Synergistic therapy, Host-guest interactions

## Abstract

**Background:**

Cancer is the most serious world's health problems on the global level and various strategies have been developed for cancer therapy. Pillar[5]arene-based supramolecular therapeutic nano-platform (SP/GOx NPs) was constructed successfully via orthogonal dynamic covalent bonds and intermolecular H-bonds with the assistance of glucose oxidase (GOx) and exhibited efficient targeted/synergistic chemo-chemodynamic cancer therapy.

**Methods:**

The morphology of SP/GOx NPs was characterized by DLS, TEM, SEM and EDS mapping. The cancer therapy efficinecy was investigated both in vivo and in vitro.

**Results:**

SP/GOx NPs can load drug molecules (Dox) and modify target molecule (FA-Py) on its surface conveniently. When the resultant FA-Py/SP/GOx/Dox NPs enters blood circulation, FA-Py will target it to cancer cells efficiently, where GOx can catalyst the overexpressed glucose to generate H_2_O_2_. Subsequently, the generated H_2_O_2_ in cancer cells catalyzed by ferrocene unit to form •OH, which can kill cancer cells. Furthermore, the loaded Dox molecules released under acid microenvironment, which can further achieve chemo-therapy.

**Conclusion:**

All the experiments showed that the excellent antitumor performance of FA-Py/SP/GOx/Dox NPs, which provided an new method for pillar[5]arene-based supramolecular polymer for biomedical applications.

**Graphical Abstract:**

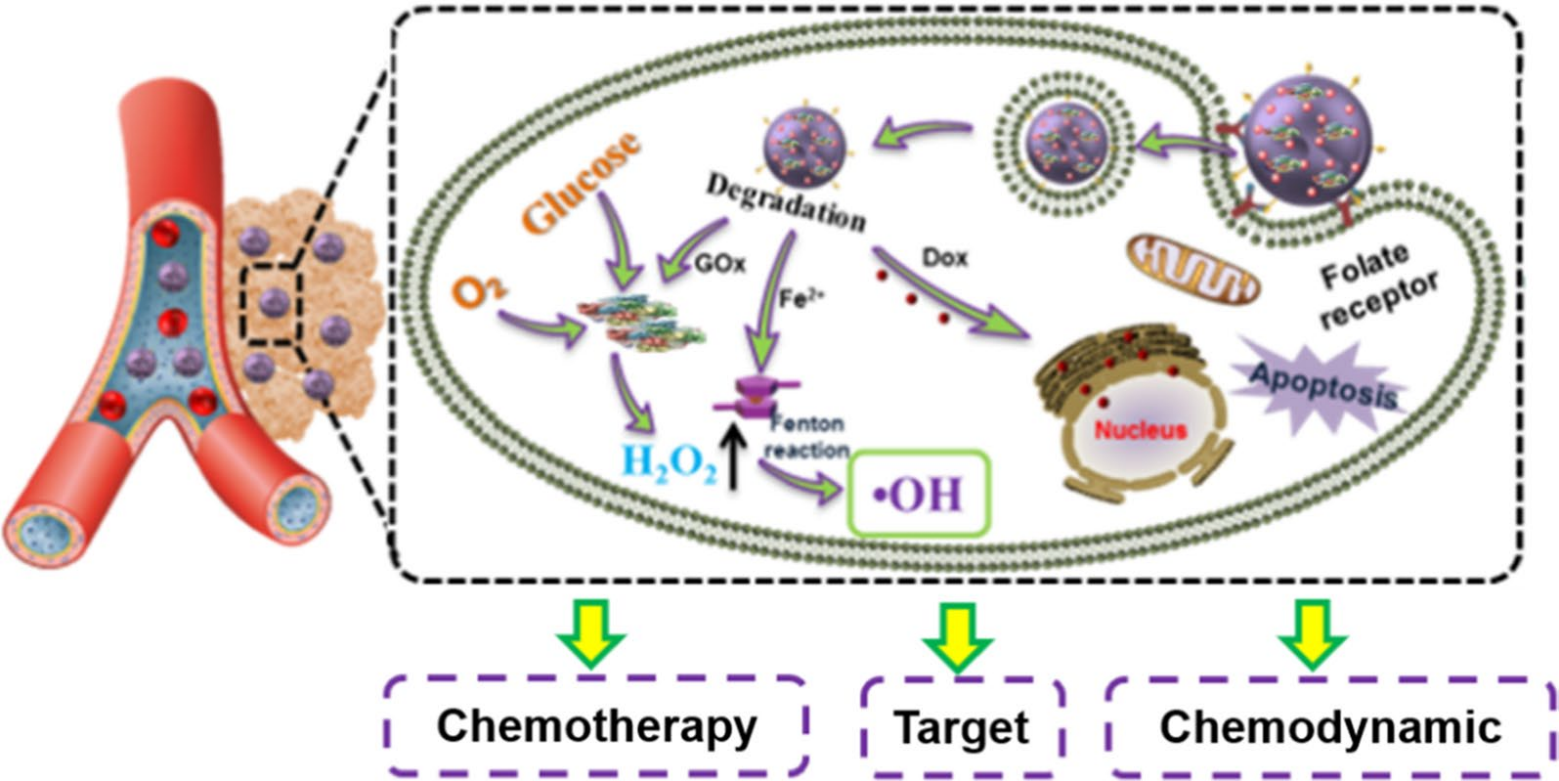

**Supplementary Information:**

The online version contains supplementary material available at 10.1186/s12951-021-01237-0.

## Background

Cancer is the most serious world's health problems on the global level and various strategies have been developed for cancer therapy [1]. During the past decade, supramolecular chemotherapy has obtained considerable interests and became an effective strategy for cancer treatment due to the dynamic and reversible nature of supramolecular chemistry endew the obtained materials with outstanding stimuli-responsibilities and infinite possibilities [2, 3] Compared with conventional chemotherapy, supramolecular chemotherapy possesses several advantages. Firstly, after forming host–guest complex or supramolecular assemblies, the solubility and stability of the anticancer drugs can be improved significantly [4]. Secondly, different functional groups, such as target units, fluorescent groups and pro-drugs, can be easily modified into systems by dynamic and reversible supramolecular interactions [5]. Thirdly, due to the differences in environments between the normal and tumor tissues, the release of the loaded drug-molecules can be precisely achieved [6]. Macrocylics, such as cyclodextrins, calixarenes and pillararenes, are the ideal platforms for construction of chemotherapeutic platforms through supramolecular interactions due to they contained size-controllable cavities where guests can be penetrated [7, 8].

Pillar[5]arenes, a new class of oligomeric macrocycles, were constructed from 1,4-dimethoxybenzene or its derivatives connected by –CH_2_– on their *p*-positions [9–11]. Compared with other classical macrocycles, such as cyclodextrins [12–15] and cucurbiturils [16, 17], the modification of pillar[5]arenes is more convenient. On the other hand, compared with crown ethers [18–20] and calixarenes [21–23]. pillar[5]arenes possess a more rigid structures and rich host–guest properties [24–37]. So, in the past 13 years, the investigation of pillar[5]arenes is becoming a research hotspot and attracted considerable interests [38–51], especially for supramolecular polymeric materials based on pillar[5]arene [52–58]. Until now, various stimuli-responsive (including light, heat, pH and so on) pillar[5]arene-based supramolecular polymers have been prepared successfully and their physicochemical properties and morphologies transformation also have been studied [59–63]. For example, Prof. Wang and *co*-workers constructed a novel pillar[5]arene-based supramolecular cross-linked polymeric pseudo[2]rotaxanes, which showed heat, pH and concentration stimuli-responsiveness [64, 65]. Recently, our group constructed a Pd nanoparticles hybrid pillar[5]arene based supramolecular, and applied it in catalytic reduction of toxic nitroaromatics and catalytic Suzuki–Miyaura reaction in water [66]. Although numerous pillar[5]arene-based supramolecular polymers and their applications have been reported, the application of them in bio-medical areas, especially in targeted and synergistic cancer therapy has rarely investigated due to their poor stability [67].

**Scheme 1.**
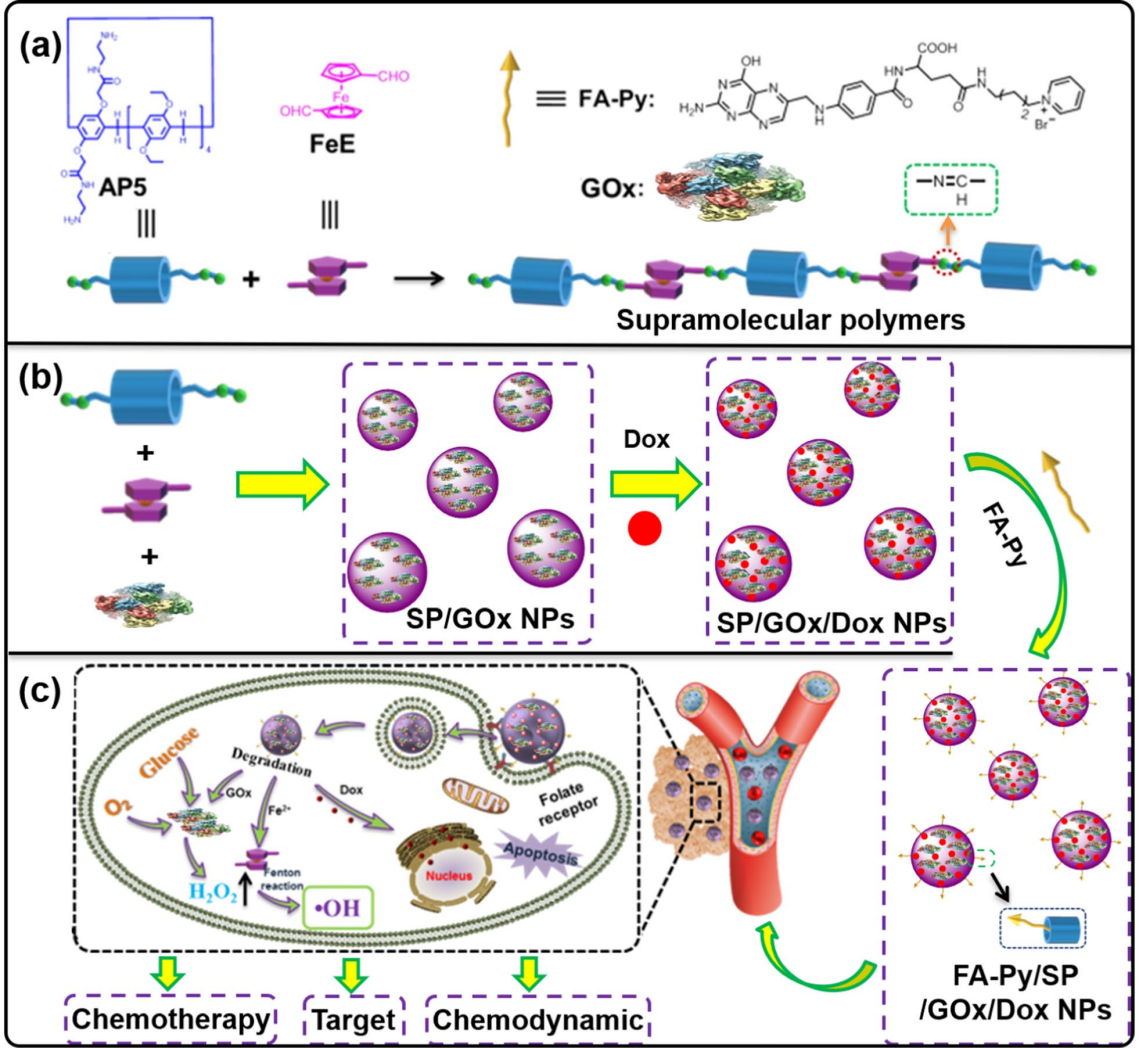
**a** Chemical structures of amino group modified pillar[5]arene (AP5), ferrocene dicarbaldehyde (FeE), and target molecule FA-Py. **b** The illustration of preparation and modification of pillar[5]arene-based supramolecular therapeutic nano-platform. **c** The targeted synergistic chemo-chemodynamic cancer therapy of the multifunctional FA-Py/SP/GOx/Dox NPs

Herein, a new pillar[5]arene-based supramolecular therapeutic nano-platform (SP/GOx NPs) was constructed from amino group modified pillar[5]arene (AP5, Scheme 1) and ferrocene dicarbaldehyde (FeE, Scheme 1) via orthogonal dynamic covalent bonds and intermolecular H-bonds with the assistance of glucose oxidase (GOx). The obtained SP/GOx NPs possess several advantages. Firstly, it can be used as drug carrier to load Dox efficiently. Secondly, the cavity of pillar[5]arene endow SP/GOx NPs with excellent host–guest properties, so target molecules (FA-Py) can be introduced on its surface easily. In this case, when the resultant pillar[5]arene-based FA-Py/SP/GOx/Dox NPs enters blood circulation, FA-Py will target it to cancer cells efficiently. Subsequently, GOx catalyst the over expression of glucose in cancer cells to form H_2_O_2_ continuously, then bridged ferrocene unit will catalysis the H_2_O_2_ to generate •OH, to achieve chemodynamic therapy. Furthermore, the loaded Dox molecules will be released under low pH micro-environment in tumor issues, which will achieve synergistic chemotherapy. In vitro experiments showed that the excellent antitumor performance of FA-Py/SP/GOx/Dox NPs, which provided an new method for pillar[5]arene-based supramolecular polymer for biomedical applications.

## Materials and methods

### Synthesis of AP5 (See Additional file 1)

#### Construction of FA-Py/SP/GOx/Dox NPs

For the synthesis of SP/GOx NPs, AP5 (9.8 mg, 0.01 mmol), FeE (5.0 mg, 0.020 mmol), and GOx (10 mg) were dissolved in the methanol/acetone mixture and then stirred at 60 °C overnight under N_2_. The SP/GOx NPs was obtained after washing with DMF, and H_2_O for several times via centrifugation (8,000 rpm × 8 min). Then SP/GOx NPs was added to the aqueous solution of Dox and stirred overnight to obtain Dox loaded materials (SP/GOx/Dox NPs). At last, SP/GOx/Dox NPs was added to the solution of FA-Py overnight to get resultant materials FA-Py/SP/GOx/Dox NPs.

#### Host–guest interaction

To determine the host–guest interaction for the complexation between AP5 and target molecule FA-Py, NMR titrations were done with solutions which had a concentration of 4.0 mM of G upon different concentration of AP5.

#### Dox loading and release

To prepare Dox-loaded SP/GOx NPs, SP/GOx NPs was added into the solution of Dox and then stirred 24 h. Then, the SP/GOx/Dox NPs was separated by washing with DMF and water 5 times and further centrifuging. Due to Dox contains a characteristic absorption at 490 nm under UV–vis spectrometer, the Dox loading capacity can be calculated by UV–vis spectrum: Dox loading capacity = (W_initial Dox_ – W_Dox in supernatant_)/W_SP/GOx NPs_ (mg mg^−1^).

The Dox release was carried out by UV–vis spectrum in different pH values (7.0, 6.0, 4.8 and 3.5) of PBS solution.

#### In vivo antitumor efficacy

When the tumor volume grew to about 100 mm^3^, all the mice were randomly divided into 7 groups (n = 5) and intravenously injected with Control, SP NPs, SP/GOx NPs, FA-Py/SP/GOx NPs, SP/Dox NPs, FA-SP/Dox NPs, and FA-Py/SP/GOx/Dox NPs for every three days. The tumor volume and body weight of the mice were monitored. The normal organs and tumor tissues were collected and investigated by H&E staining at 21th day.

## Results

### Preparation and characterization of SP/GOx NPs

Amino-amide group modified pillar[5]arene (AP5) was designed and prepared in two steps. As shown in Scheme S1, copillar[5]arene **3** was first prepared from condensation of monomer **1** and **2** (**1**: **2** = 1: 4) with BF_3_•Et_2_O as the catalyst. Then AP5 was obtained by refluxing a solution of **3** and excess 1,2-ethanediamine in ethanol. With AP5 in hands, supramolecular polymeric nanoparticles with (SP/GOx NPs) or without (SP NPs) GOx were constructed in DMSO at 80 ˚C overnight by employing AP5 and ferrocene dicarbaldehyde (FeE) as building blocks. In order to investigate the change of AP5 and FeE during the construction of polymers, ^1^H NMR of the chemical species against time was performed. As shown in Additional file 1: Fig. S6, the proton of the aldehyde groups on FeE became smaller and smaller as the reaction proceeds, and disappeared after 40 min (Additional file 1: Fig. S6e), indicating that the carbazone reaction was completed.

**Fig. 1:**
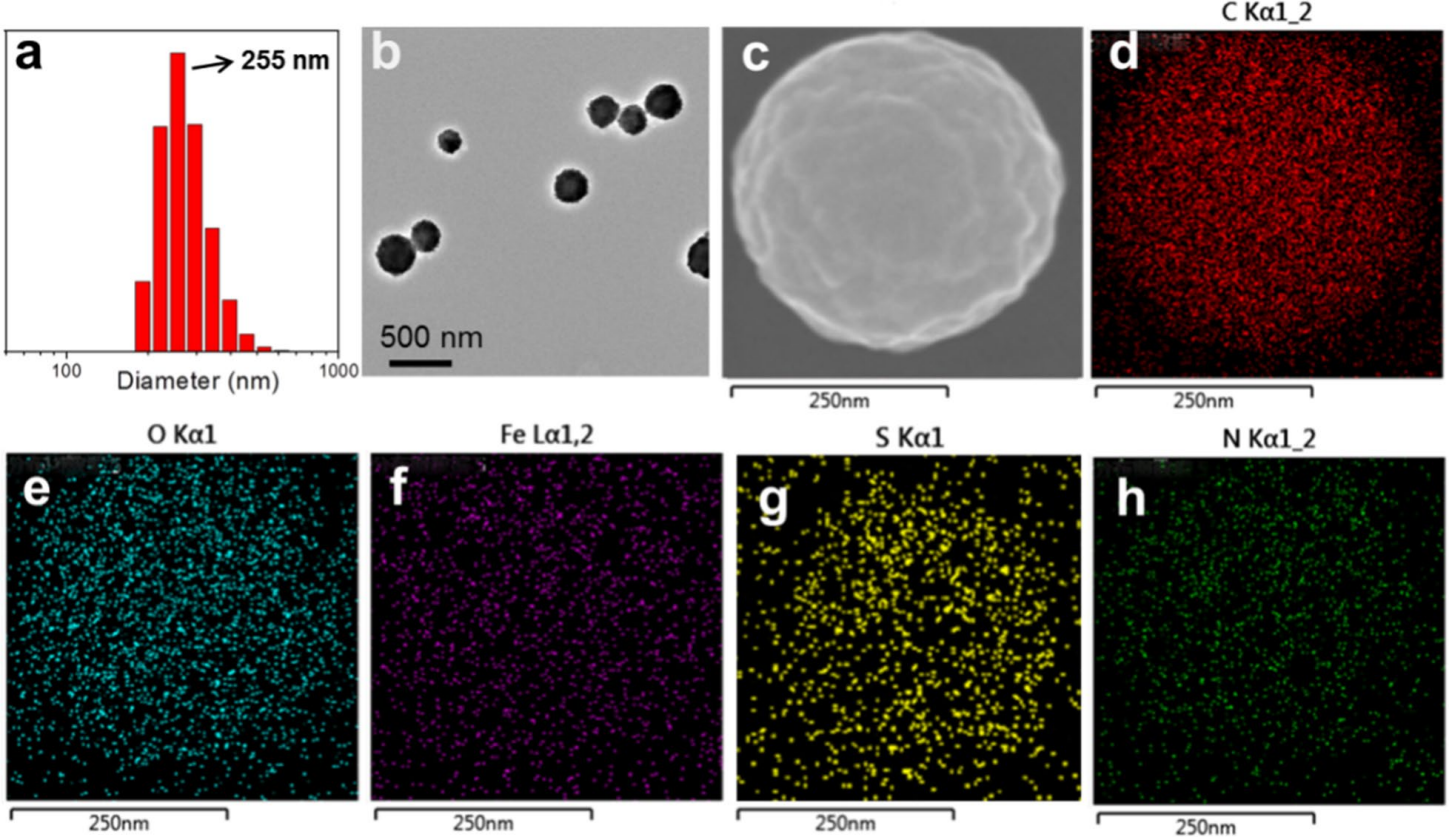
**a** DLS study of SP/GOx NPs dispersed in water. **b** TEM image and **c** Enlarged SEM image of SP/GOx NPs. **d**–**h** EDS mapping images of SP/GOx NPs

The morphology of SP/GOx NPs was then characterized by dynamic light scattering (DLS), transmission electron microscopy (TEM) and scanning electron microscopy (SEM) images. As shown in Fig. 1a, DLS study showed that SP/GOx NPs was mono-dispersed in water with an average diameter about 255 nm. From TEM image, we found that SP/GOx NPs was in uniform spherical structure and the diameter distribute from 200 to 350 nm (Fig. 1b), which consist with the DLS investigation. Furthermore, the chemical composition of SP/GOx NPs was revealed by energy dispersive X-ray spectroscopy (EDS) mapping. As shown in Fig. 1d–h, C, O, Fe, S, N elements were dispersed homogeneously in SP/GOx NPs, demonstrating they are from AP5, FeE and GOx.

### Chemodynamic performance of SP/GOx NPs

**Fig. 2:**
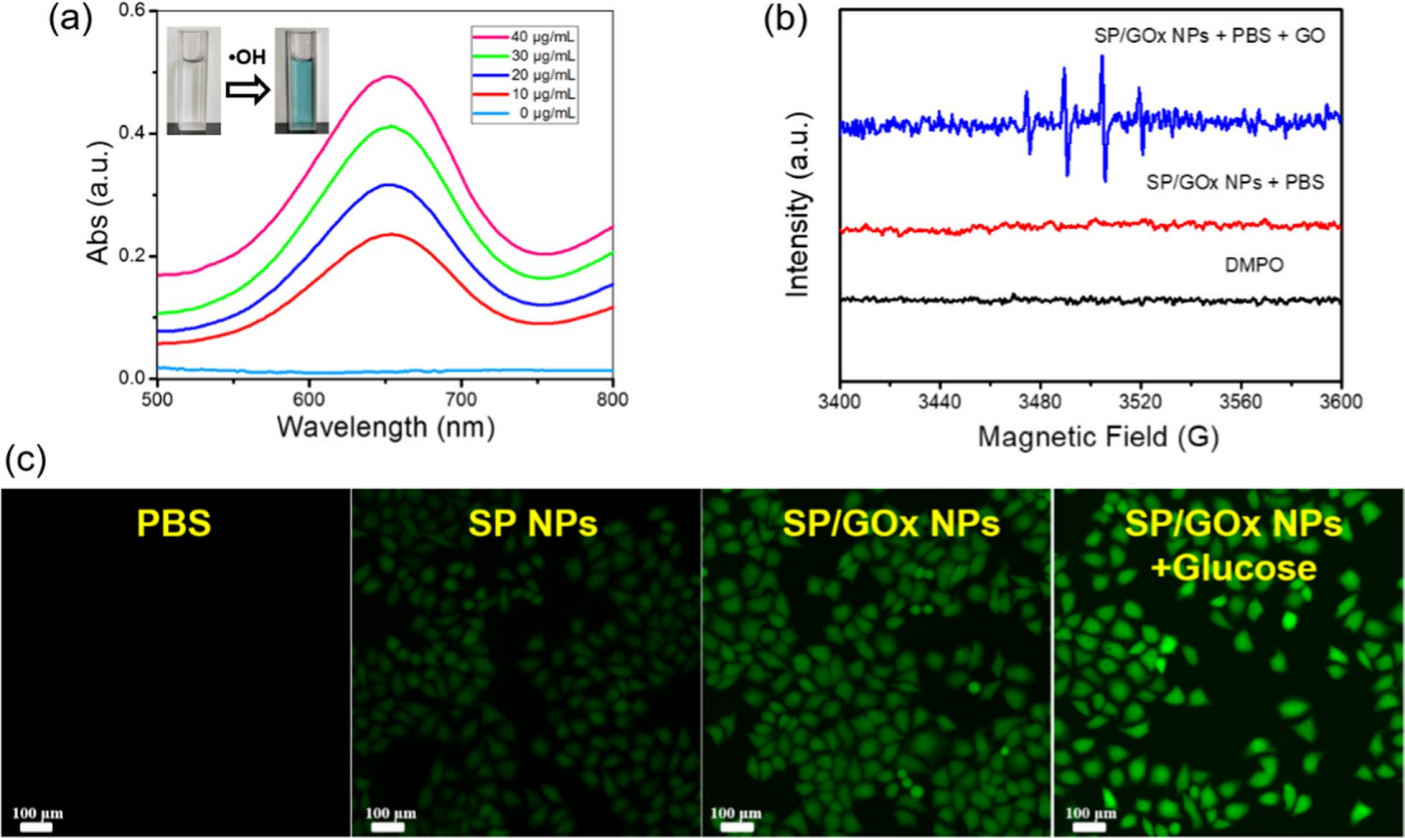
**a** Time-dependent UV–visible spectra of the solution containing SP/GOx NPs, H_2_O_2_, and TMB ([TMB] = 0.25 mmol, [H_2_O_2_] = 1.00 mmol, SP/GOx NPs: 0.15 mg). **b** Electron spin resonance spectra of •OH trapped by DMPO in SP/GOx NPs + PBS + GO (blue line), SP/GOx NPs + PBS (red line) and PBS (black line). **c** CLSM image of •OH generation in HeLa cells after different treatments with DCFH-DA as the probe

As we all known that Fe^2+^ ion is a classical Fenton-like catalyst, which can catalysis H_2_O_2_ to generate •OH to kill cancer cells in tumor issues and to achieve chemodynamic therapy (CDT) [68]. Here, we used 3,3’,5,5’-tetramethylbenzidine (TMB) to detect the •OH generation ability of SP/GOx NPs due to non-fluorescent TMB can be oxidized by •OH to form fluorescent ox-TMB. As shown in Fig. 2a, in the absence of SP/GOx NPs, there was no absorbance peak of the solution of H_2_O_2_ & TMB (light blue line). However, upon addition of SP/GOx NPs, the absorbance peak at 650 nm was observed (red line), suggesting the generation of •OH. The more SP/GOx NPs added, the faster •OH produced (Fig. 2a, blue/green/pink line). Furthermore, electron paramagnetic resonance (EPR) spectra were performed to verify the formed •OH with 5,5-dimethly-1-proline-N-oxide (DMPO) as the detector [69]. In the GO’ PBS solution, •OH signal can be found clearly upon addition of SP/GOx NPs, as shown in Fig. 2b (blue line). For comparison, if there is no GO in the PBS solution, no •OH was generated (Fig. 2b, red line). The intracellular •OH generation in HeLa cells is further confirmed by adopting DCFH-DA as the fluorescence indicator. As shown in Fig. 2c, in PBS group, HeLa cells showed no fluorescence, while in SP NPs group, week green fluorescence was observed due to the Fenton activity by consuming the endogenous H_2_O_2_. However, when HeLa cells are incubated with SP/GOx NPs, strong green emission was observed. This is due to GOx can catalyst the over-expressed GO in cancer cells to form H_2_O_2_, which then further catalyst by Fe^2+^ through Fenton reaction to generate •OH continuously.

### Cytotoxicity of FA-Py/SP/Gox/Dox NPs toward cancer cells

**Fig. 3:**
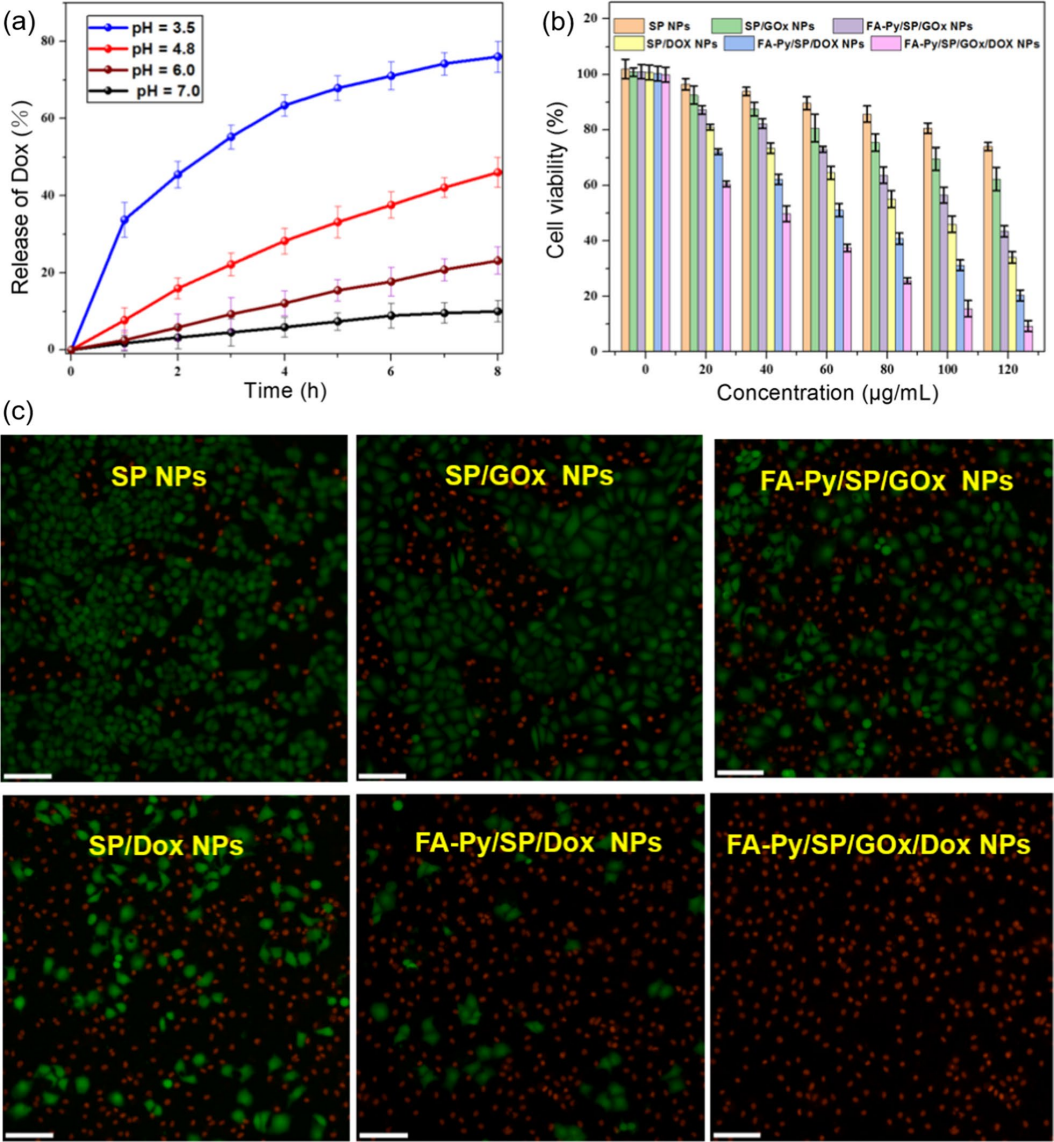
**a** Dox release profiles from SP/GOx NPs at pH 3.5, pH 4.8, pH 6.0 and pH 7.4, respectively. **b** Cell viabilities of HeLa cells incubated with SP NPs, SP/GOx NPs, FA-Py/SP/GOx NPs, SP/Dox NPs, FA-Py/SP/Dox NPs, and FA-Py/SP/GOx/Dox NPs at different concentrations. **c** Fluorescence images of Calcein AM (live cells, green) and PI (dead cells, red) costained HeLa cells after different groups

In the process of traditional tumor chemotherapy, the poor water solubility and high toxicity of drugs greatly inhibit the therapeutic effect. In this work, in order to further explore the potential application of the obtained SP/GOx NPs as a drug carrier, the drug loading and in vitro release was carried out with Doxorubicin hydrochloride (Dox) as the drug molecule. As shown in Additional file 1: Fig. S7, after adding 9.5 mg SP/GOx NPs into the solution contain 4.5 mg Dox and stirred 24 h, the color of the solution became weak, indicating that Dox was loaded by SP/GOx NPs. Finally, it was calculated that approximately 3.74 mg Dox was adsorbed and loaded in SP/GOx NPs (394 µg/mg). The successful preparation of SP/GOx/Dox NPs was also confirmed by UV and FT-IR spectra (Additional file 1: Fig. S13). The release behaviors of SP/GOx/Dox NPs were evaluated in PBS with pH 7.0, 6.0, 4.8, and 3.5, respectively (Fig. 3a). The real-time drug release profiles of Dox were monitored by UV–vis spectrophotometry. After 8 h, the cumulative release rate reached 10.0% at pH 7.0, 23.2% at pH 6.0, 46.1% at pH 4.8, and 76.1% at pH 3.5, suggesting that SP/GOx/Dox NPs can be used as a sustained release carrier for cancer therapy.

More importantly, besides applied as drug carrier, SP/GOx NPs can further modified with targeted molecules (FA-Py) onto its surface to form FA-Py/SP/GOx/Dox NPs due to its internal host–guest interaction of pillar[5]arene framework (Additional file 1: Fig. S8 and S9), thus achieving targeted treatment of tumors. In vivo imaging of the biodistribution of the SP/Dox NPs and FA-Py/SP/Dox NPs clearly confirmed the tumor targeting ability after FA-Py modification (Additional file 1: Fig. S12). Furthermore, confocal laser scanning microscope (CLSM) was used to investigate the cellular uptake of the resultant SP/GOx/Dox NPs and FA-Py/SP/GOx/Dox NPs by Hela cells. As shown in Additional file 1: Fig. S10, the nucleus were stained by blue fluorescent DAPI to locate the predetermined cells. When treated with FA-Py/SP/GOx/Dox NPs, the cells exhibited more bright red fluorescence than that treated with SP/GOx/Dox NPs under the same conditions, indicating that FA-Py can target the materials to cancer cell efficiently. In addition, the merge image of NPs clearly showed that after 4 h of incubation, the fluorescence intensity of the cells was higher than that of those treated for 1 h, confirming that cellular uptake of FA-Py/SP/GOx/Dox NPs was a time-dependent process. To investigate the in vitro targeted and combined chemo-chemodynamic cancer therapy of the resultant FA-Py/SP/GOx/Dox NPs, HeLa cells was selected, and its viability after treating with different groups was also investigated via 3-(4,5-dimethylthiazol-2-yl)-2,5-diphenyltetrazolium bromide (MTT) assays. Firstly, cells were incubated with various concentrations (0–120 μg/mL) of SP NPs, SP/GOx NPs, FA-Py/SP/GOx NPs, SP/Dox NPs, FA-Py/SP/Dox NPs, and FA-Py/SP/GOx/Dox NPs.

As shown in Fig. 3b, SP NPs exhibit high cell viability (80%) even when the concentration is 120 µg/mL due to only over expressed H_2_O_2_ in tumor issues transformed into •OH by the contained ferrocene units. SP/GOx NPs showed lower cell viability than SP NPs under the same conditions due to GOx can catalyst the over expressed GO in tumor cells into H_2_O_2_ continuously. For FA-Py/SP/GOx NPs, FA-Py molecule can target the particles to cancer cells efficiently, so the cell viability further decreased. Importantly, after loading Dox, all the groups exhibited higher cytotoxicity, especially for the resultant FA-Py/SP/Gox/Dox NPs, the cell viability decreased to 6% when its concentration increased to 120 μg/mL. Furthermore, to visualize the targeted and combined chemo-chemodynamic cancer therapy effect of FA-Py/SP/GOx/Dox NPs, calceinacetoxymethyl (Calcein-AM) and propidium iodide (PI) staining was employed to differentiate dead (red) and live (green) cells (Fig. 3c). The cells treated with SP NPs showed almost bright green fluorescence, indicating they lived well. Nevertheless, cells incubated with FA-Py/SP/GOx/Dox NPs were all died and exhibited red fluorescence. All the above results confirmly certified the excellent therapeutic effect of FA-Py/SP/GOx/Dox NPs.

### In vivo targeted chemo-chemodynamic therapy efficacy of FA-Py/SP/GOx/Dox NPs

**Fig. 4:**
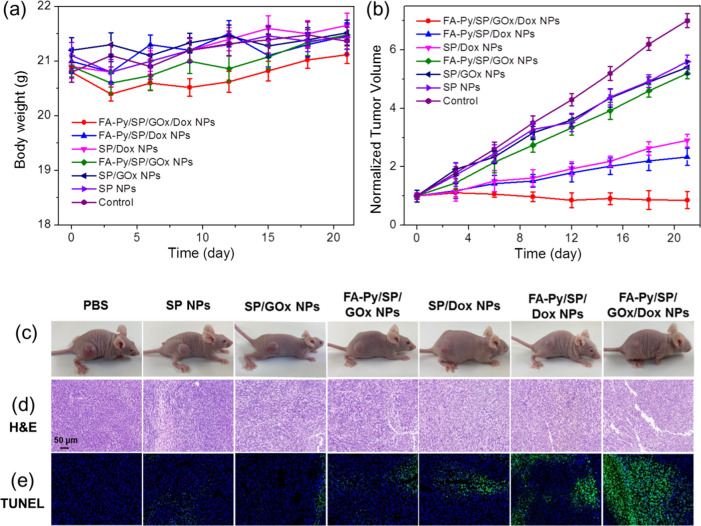
**a** Body weight curves and **b** normalized tumor volume curves of tumor-bearing KM mice in different treatment groups. **c** Representative tumor photograph at 21th day. **d** H&E and **e** TUNEL staining of tumors in corresponding groups

Inspired by the outstanding therapeutic effect of FA-Py/SP/GOx/Dox NPs in vitro, the in vivo antitumor efficacy on mouse was also performed. Firstly, the mice were divided into 7 groups (Control, SP NPs, SP/GOx NPs, FA-Py/SP/GOx NPs, SP/Dox NPs, FA-SP/Dox NPs, and FA-Py/SP/GOx/Dox NPs) randomly when the size of tumors reached ~ 100 mm^3^. As shown in Fig. 4a, there was no significant change in body weight in all groups during the treatment period, indicating that the this pillar[5]arene based nanomaterials are reasonably safe. Then the tumor volumes of mice were monitored every three days to check the cancer therapy activity of different groups. As shown in Fig. 4b, the tumor volume increased rapidly for the mice in Control group, while the SP NPs, SP/GOx NPs and FA-Py/SP/GOx NPs showed a slight tumor suppressive effect. However, The cancer therapy efficacy of SP/Dox NPs was better than those of SP NPs but lower than the FA-Py/SP/Dox NPs, demonstrating the important role of the target molecule FA-Py. Significantly, FA-Py/SP/GOx/Dox NPs showed the best inhibitory effect to tumor, indicating the excellent therapeutic effect of the targeted and combined chemo-chemodynamic cancer therapy.

The photograph of the tumors treated with different treatments was observed in Fig. 4c. The Control group had the largest tumor, followed by SP NPs group, SP/GOx NPs group, and FA-Py/SP/GOx NPs group. SP/Dox NPs group and FA-Py/SP/Dox NPs group were smaller. However, FA-Py/SP/GOx/Dox NPs group showed the smallest tumor, which consist with the above tumor volume results. Furthermore, from H&E and TUNEL staining assay (Fig. 4d, e) we found that the FA-Py/SP/GOx/Dox NPs group showed the most prominent reduction in tumor cells, and the most TUNEL-positive tumor cells. Then in order to investigate the side effects on the major organs of the mice, the H&E staining of heart, liver, spleen, lung, and kidney were performed, which confirmed that no obvious inflammatory damage and tissue damage in all major organs (Additional file 1: Fig. S11). All the results certified that this pillar[5]arene based supramolecular materials possess excellent efficiency for cancer therapy with good biocompatibility.

## Conclusion

In conclusion, pillar[5]arene-based supramolecular polymeric materials (SP/GOx NPs) was prepared from AP5 and FeE with the assistant of GOx. NMR, SEM, TEM and EDS-Mapping were applied to characterize its morphology and chemical compositions. The obtained SP/GOx NPs can load drug molecules (Dox) and modify target molecule (FA-Py) on its surface due to it possess both the advantages of adsorption ability and rich host–guest interaction. In this case, when the resultant FA-Py/SP/GOx/Dox NPs enters blood circulation, FA-Py will target it to cancer cells efficiently, where GOx can catalyst the overexpressed GO to generate H_2_O_2_. Subsequently, the generated H_2_O_2_ in cancer cells catalyzed by ferrocene unit to form •OH, which can kill cancer cells. Furthermore, the loaded Dox molecules released under acid microenvironment, which can further achieve chemo-therapy. Both the in vitro and in vivo investigations certified that the excellent targeted and combined chemo-chemodynamic cancer therapy of Py/SP/GOx/Dox NPs.

## Supplementary Information

**Additional file 1.** Materials. Characterization. Synthesis of AP5. Construction of FA-Py/SP/GOx/Dox NPs. Cell experiments. Animal experiments.
