# Supplementary material for: GOx-assisted synthesis of pillar[5]arene based supramolecular polymeric nanoparticles for targeted/synergistic chemo-chemodynamic cancer therapy
Source: J Nanobiotechnology. 2022 Jan 11;20:33. doi: 10.1186/s12951-021-01237-0 (PMC8753913; doi:10.1186/s12951-021-01237-0)
Supplement: Supplementary file 1 — Additional file 1. Materials. Characterization. Synthesis of AP5. Construction of FA-Py/SP/GOx/Dox NPs. Cell experiments. Animal experiments. [file 12951_2021_1237_MOESM1_ESM.docx]

Additional file 1

GOx-Assisted Synthesis of Pillar[5]arene Based Supramolecular Polymeric Nanoparticles for Targeted/Synergistic Chemo-Chemodynamic Cancer Therapy

Jin Wang,^†^ Di Wang,^†^ Moupan Cen, Danni Jing, Jiali Bei, Youyou Huang, Jianan Zhang, Bing Lu, Yang Wang,* and Yong Yao*

1. Materials…………………………………………………………………………………S2
2. Characterization………………………………………………………………………...S2
3. Synthesis of AP5…………………………………………………………………………S2
4. Construction of FA-Py/SP/GOx/Dox NPs……………………………………………..S5
5. Cell experiments…………………………………………………………………………S8
6. Animal experiments……………………………………………………………………..S9
7. **Materials**

All chemical reagents are of analytical grade and are not further purified. Specifically, BF_3_•Et_2_O, ClCH_2_COOCH_3_, NH_2_CH_2_CH_2_NH_2_, CH_3_CH_2_OH, (CH_2_O)n, hydroquinone, ferrocene dicarbaldehyde were purchased from Shanghai Aladdin Industrial Corporation. Doxorubicin (DOX) and folic acid (FA) were purchased from Sinoreagent Co. Ltd. All the solutions were prepared in deionized water or phosphate buffer saline (PBS, Gibco). The Cell Counting Kit (CCK)-8 was bought from MedChemExpress Co. Ltd.

**2. Characterization**

The morphology and microstructure were obtained by field emission scanning electron microscopy (SEM) and transmission electron microscopy (TEM). SEM was performed by using a HITACHI S-4800 microscope at an accelerating voltage of 5 kV. TEM observations were performed by a Philips CM20. The N_2_ adsorption desorption curves were recorded on a Quad-resorb-SI instrument and the specific surface area was examined by the Brunauer-Emmett-Teller (BET) process. ^1^H and ^13^C NMR spectra were recorded on a Brucker AV400 spectrometer. UV/Vis spectra and the optical transmittance were recorded in a quartz cell (light path 10 mm) on a Shimadzu UV-3600 spectrophotometer equipped with a PTC-348WI temperature controller. Electrospray ionization mass spectra (ESI-MS) were measured by Agilent 6520 Q-TOF-MS.

**3. Synthesis of AP5**

Scheme S1. Synthetic route to compound AP5.

A solution of **1** (2.54 g, 10.0 mmol), **2** (6.64g, 40.0 mmol) and triformol (1.50 g, 50.0 mmol) in 1,2-dichloroethane (50 mL) was cooled with ice bath. Boron trifluoride etherate (16.0 g, 50.0 mmol) was added to the solution and the mixture was stirred at room temperature for 1 hour. The reaction mixture was then washed by water (50 mL × 2) and dried with Na_2_SO_4_. The solvent was evaporated to provide a crude product, which was purified by column chromatography (eluent: petroleum ether/ethyl acetate, 5:1) to give a white solid **3** (4.58 g, 47%). ^1^H NMR (400 MHz, CDCl_3_) was shown in Figure S1, δ : 6.83 – 6.76 (m, 8H), 4.51 (s, 4H), 3.88 (ddd, J = 13.5, 11.4, 6.7 Hz, 16H), 3.80 – 3.73 (m, 10H), 3.13 (s, 6H), 1.37 (ddt, J = 13.9, 10.6, 5.4 Hz, 24H). ^13^C NMR (101 MHz, CDCl_3_) was shown in Figure S2, δ: 169.8, 149.7, 149.6, 149.6, 149.4, 128.8, 128.6, 128.4, 128.3, 127.7, 115.1, 114.6, 114.5, 114.4, 77.4, 77.0, 76.7, 65.8, 63.8, 63.6, 63.5, 51.6, 29.7, 29.5, 29.4, 15.3, 15.2. HRESIMS: m/z calcd for [1 + Na]^+^ 1001.4605; found 1001.4605.

A mixture of **3** (0.98 g, 1.00 mmol) and ethanediamine (5 mL) in ethanol (25 mL) was stirred in a 100 mL round-bottom flask at 90 ºC for 24 hours. After cooling, the solvent was removed and the residue was poured into saturated brine (50 mL) to give **AP5** as a white solid (0.78 g, 75%). ^1^H NMR (400 MHz, CDCl_3_) was shown in Figure S3, δ 6.97 (s, 2H), 6.94 (s, 2H), 6.89 (s, 2H), 6.87 (s, 2H), 6.73 (s, 2H), 4.52 (s, 4H), 3.92 (dq, *J* = 19.7, 6.9 Hz, 16H), 3.77 (d, *J* = 7.6 Hz, 10H), 2.39 (s, 2H), 1.99 (d, *J* = 17.6 Hz, 2H), 1.48 (dt, *J* = 24.6, 6.9 Hz, 28H). ^13^C NMR (101 MHz, CDCl_3_) was shown in Figure S4, δ: 167.0, 148.9, 148.7, 148.2, 147.4, 128.5, 128.0, 127.0, 126.0, 123.8, 113.9, 113.6, 113.4, 113.3, 112.6, 76.3, 76.0, 75.7, 66.8, 63.1, 62.9, 62.4, 40.2, 38.7, 37.8, 28.4, 27.5, 14.5, 14.4, 14.3. HRESIMS: m/z calcd for [1 + Na]^+^ 1057.5465; found 1057.5465 (Figure S5).


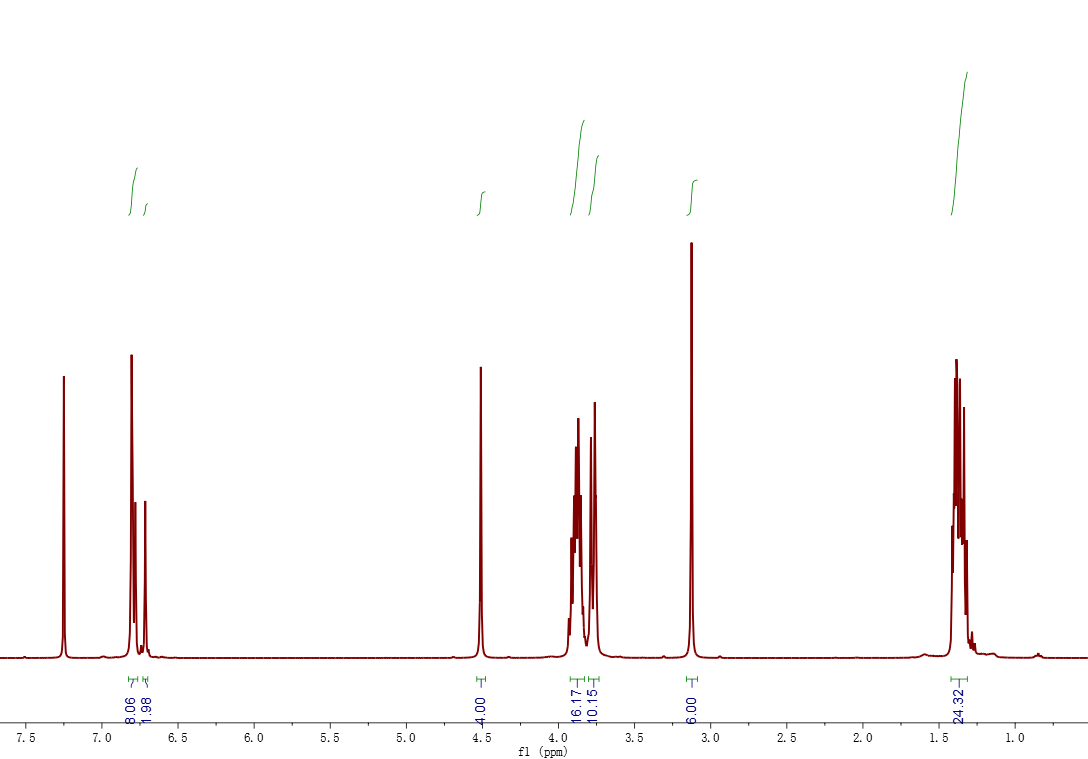


Figure S1. ^1^H NMR spectrum (400 MHz, CDCl_3_, 293 K) of **3**.


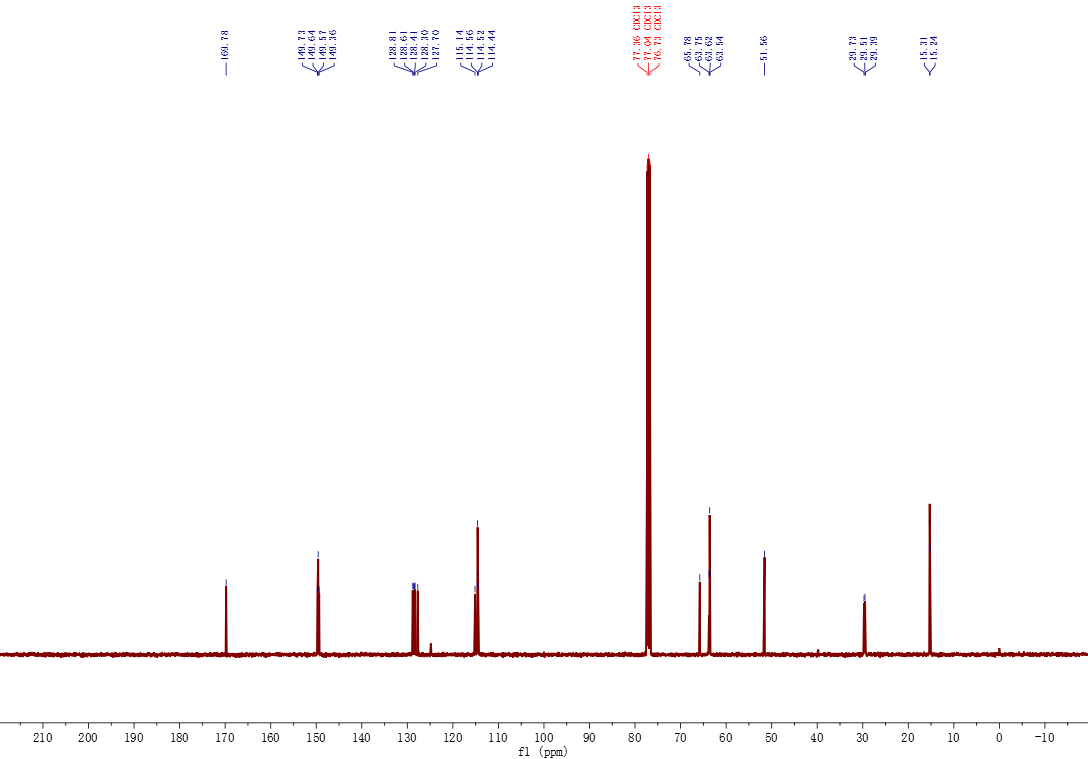


Figure S2. ^13^C NMR spectrum (100 MHz, CDCl_3_, 293 K) of **3**.


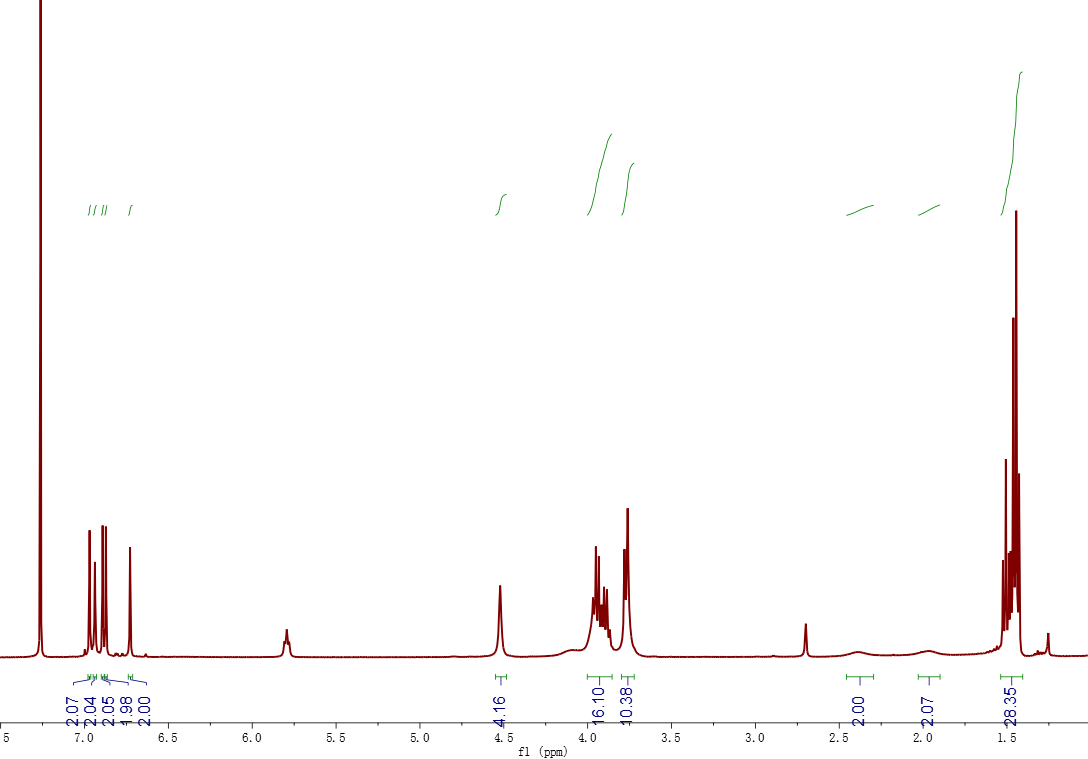


Figure S3. ^1^H NMR spectrum (400 MHz, CDCl_3_, 293 K) of AP5.


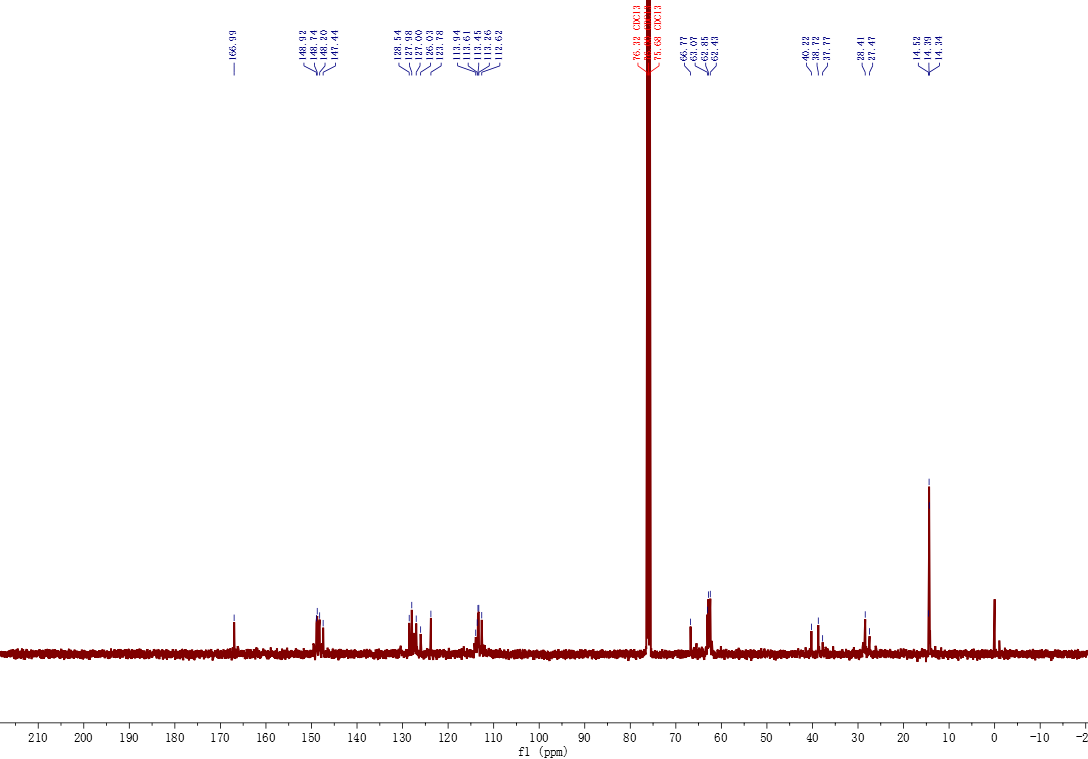


Figure S4. ^13^C NMR spectrum (100 MHz, CDCl_3_, 293 K) of AP5.


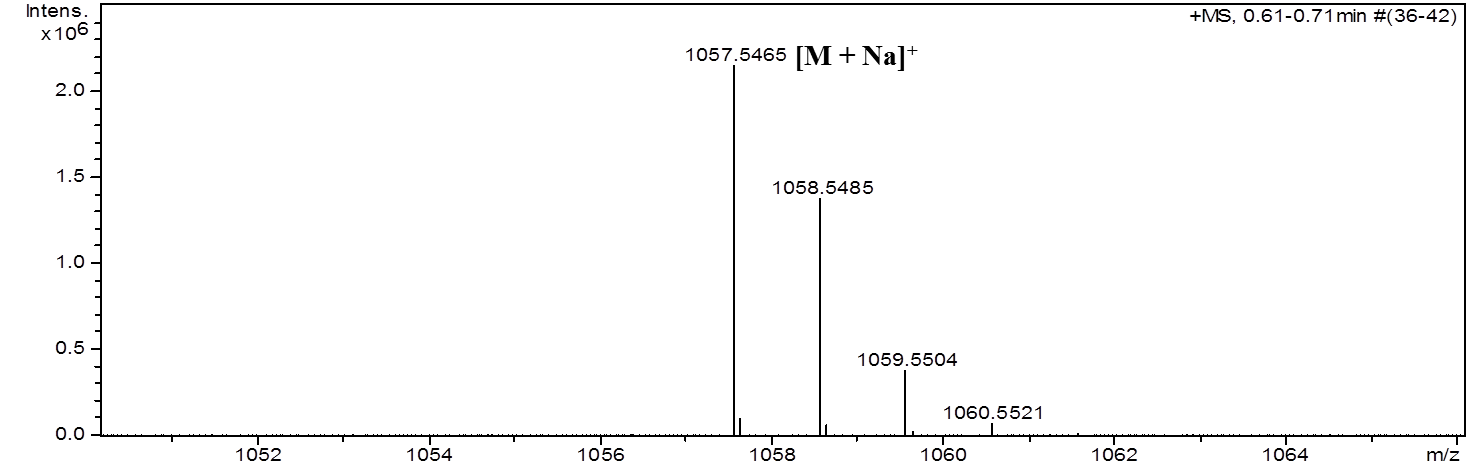


Figure S5. Electrospray ionization mass spectrum of AP5. Assignment of main peaks: *m*/*z* 1057.5465 [AP5 + Na]^+^ (100%).

**4. Construction of FA-Py/SP/GOx/Dox NPs**

For the synthesis of SP/GOx NPs, AP5 (13.5 mg, 0.02 mmol), FeE (5.6 mg, 0.027 mmol), GOx (20 mg, 34 wt% feed ratio), as well as mixed solvent of methanol (20 mL) and acetone (20 mL) were added to a flask (100 mL). The mixture was stirred at 40 °C for 24 h under nitrogen. Then, the product was obtained after washing with *N,N*-dimethylformamide, acetone and water for several times *via* centrifugation (10,000 rpm × 10 min). Then SP/GOx NPs was added to the aqueous solution of Dox and stirred overnight to obtain Dox loaded materials (SP/GOx/Dox NPs). At last, SP/GOx/Dox NPs was added to the solution of FA-Py overnight to get resultant materials FA-Py/SP/GOx/Dox NPs.


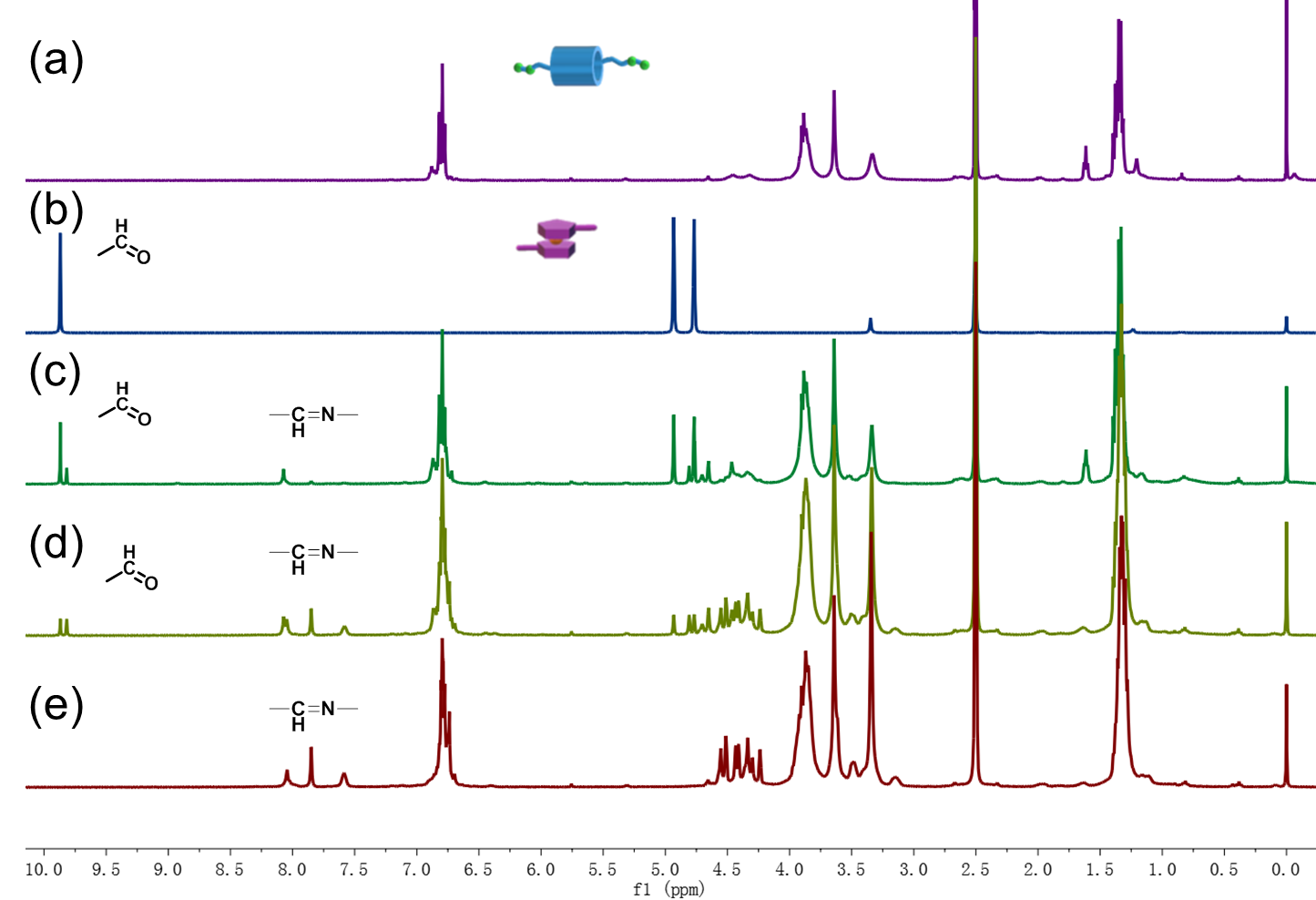


Figure S6. ^1^H NMR spectrum (400 MHz, DMSO-*d*_6_, 293 K) of (a) AP5, (b) FeE, (c) fresh (AP5 + FeE), (d) (AP5 + FeE) reacted 4 h, (e) (AP5 + FeE) reacted 24 h.


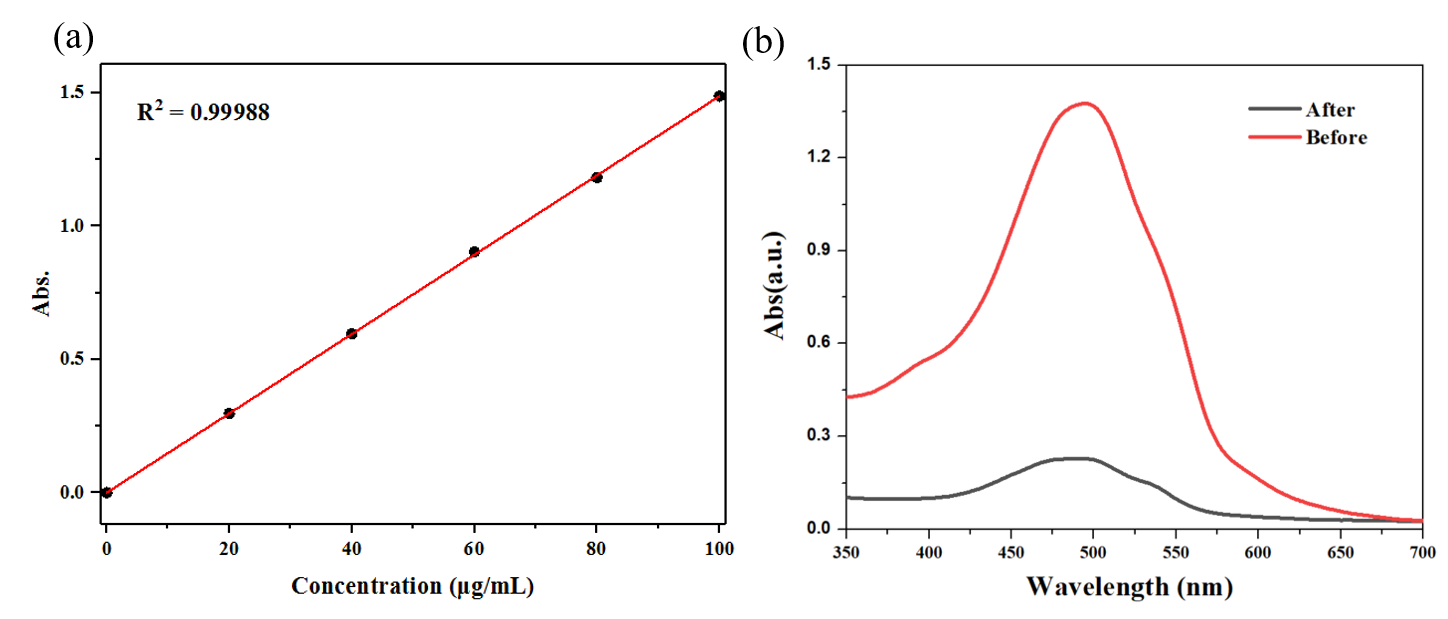


Figure S7. (a) Absorbance intensity at 480 nm as a function of the concentration of Dox in solution. (b) The UV-vis absorbance of the Dox solution before and after Dox loading.


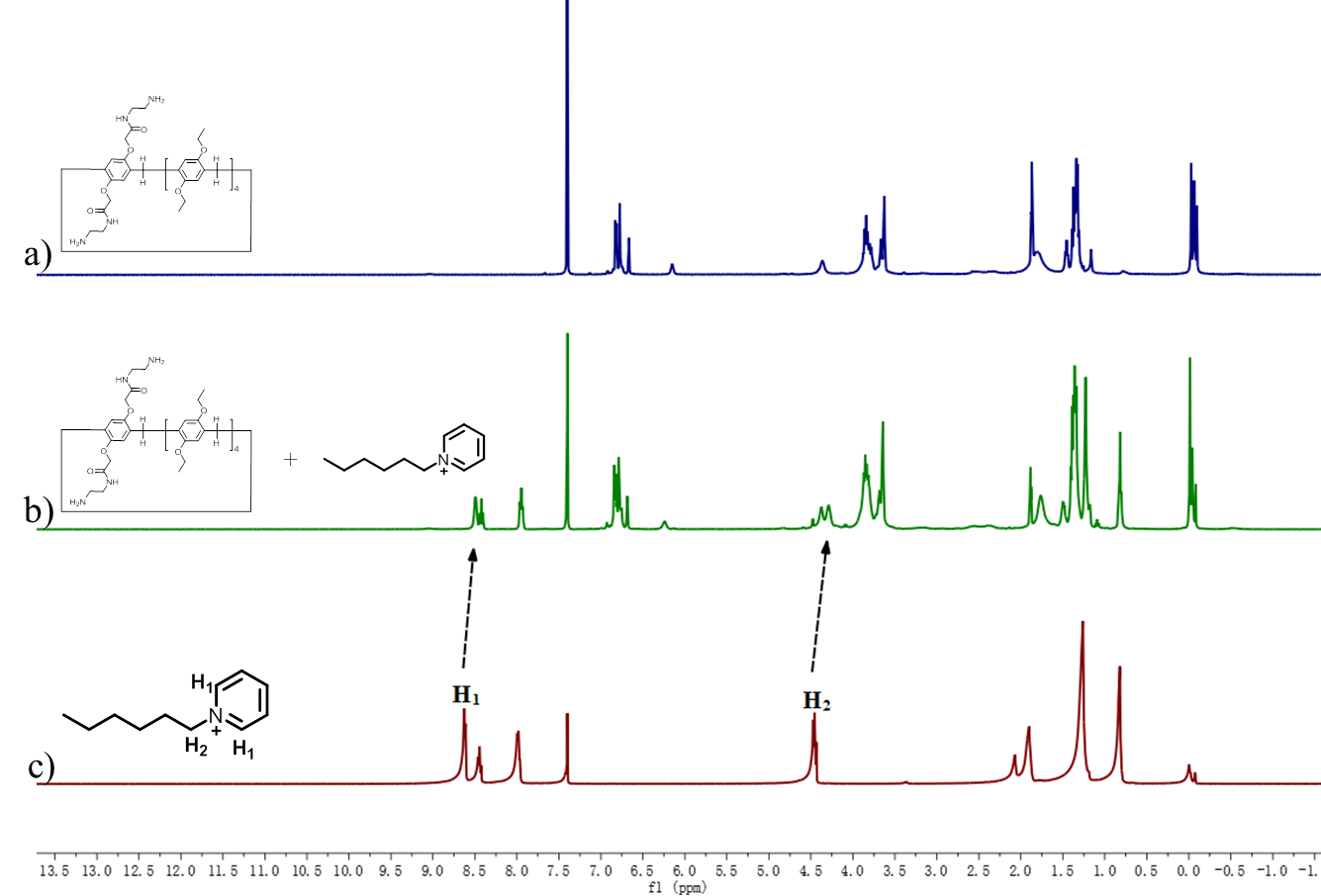


Figure S8. ^1^H NMR spectra (400 MHz, CDCl_3_/Acetonitrile-D_3_, 298 K) of (a) AP5 (10.0 mM), (b) AP5 + G ([AP5] = 10.0 mM, [G]=10.0 mM), and (c) G (10.0 mM). G is the model compound of FA-Py.


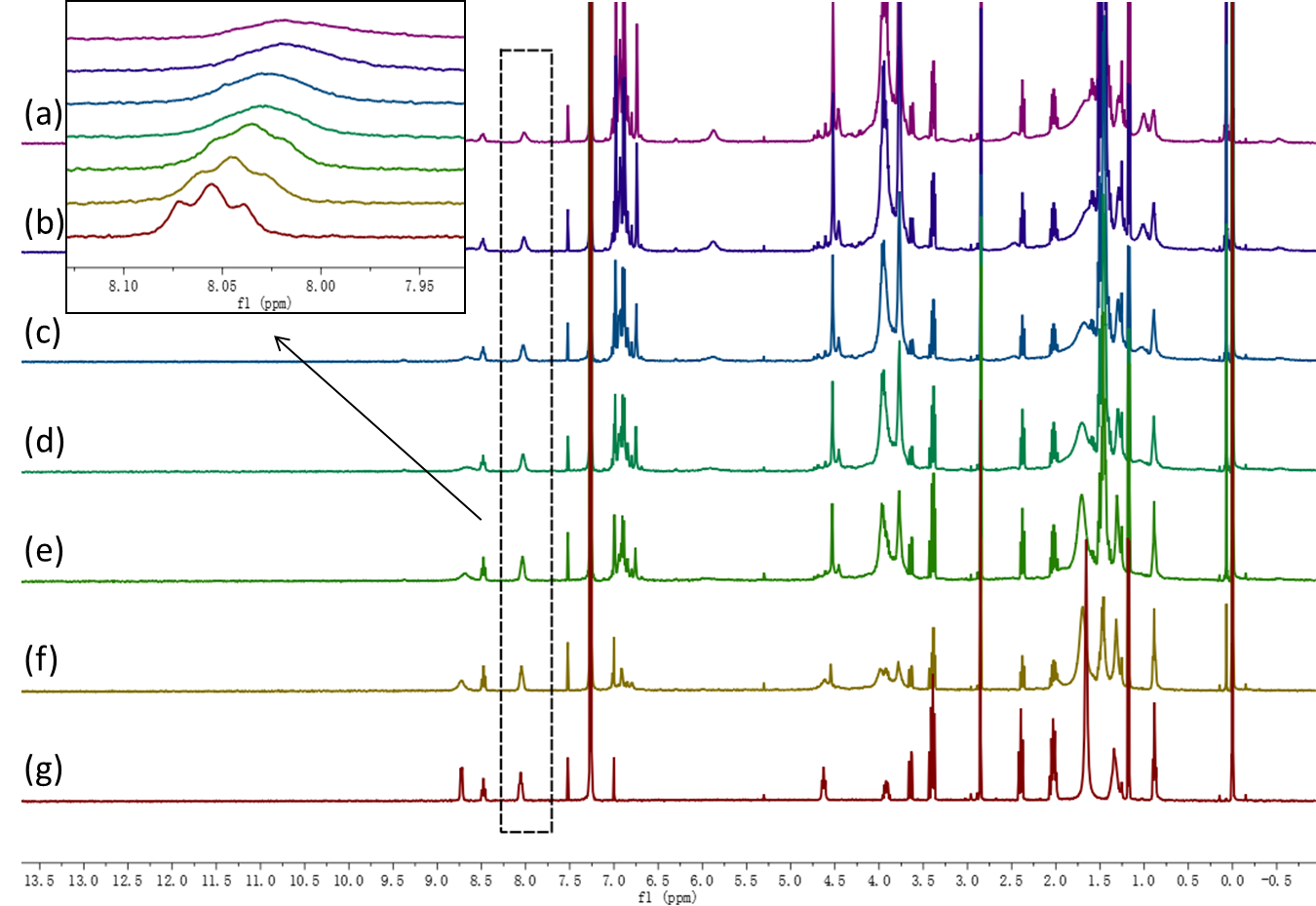


Figure S9 ^1^H NMR spectra (CDCl_3_, 293 K, 400Hz) of G at a concentration of 4.0 mM upon different concentration of AP5:(a) 0 mM, (b) 1 mM, (c) 2 mM, (d) 3 mM, (e) 4 mM, (f) 5 mM, (g) 6 mM. G is the model compound of FA-Py.

**5. Cell experiments**


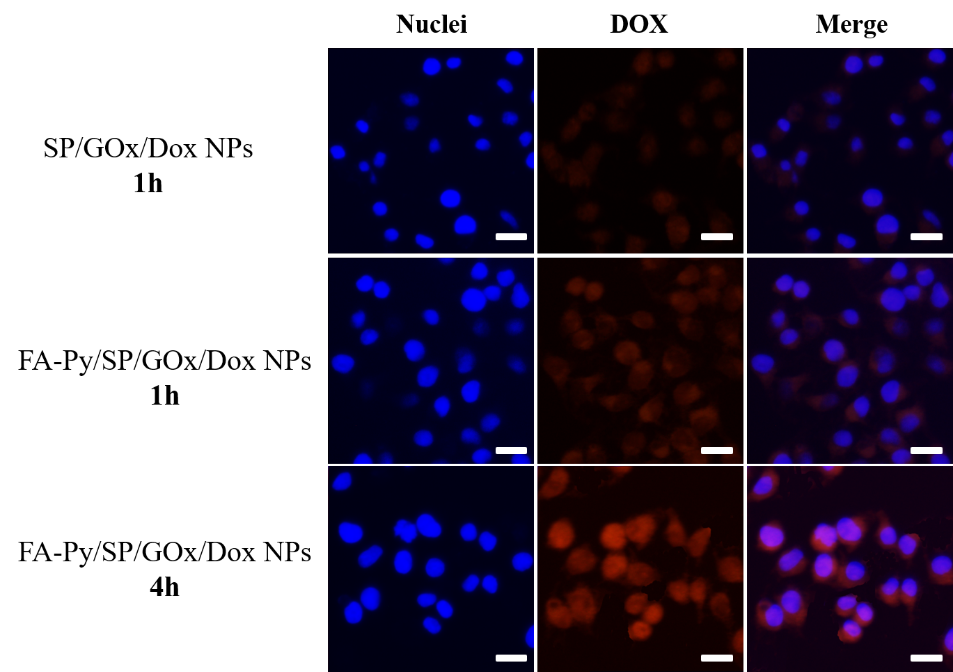


Figure S10 CLSM images of Hela cells incubated with SP/GOx/Dox NPs or FA-Py/SP/GOx/Dox NPs (100 μg/mL). The blue fluorescence of DAPI shows the location of the nuclei and the red fluorescence represents Dox. Scale bar: 50 µm.

**6. Animal experiments**


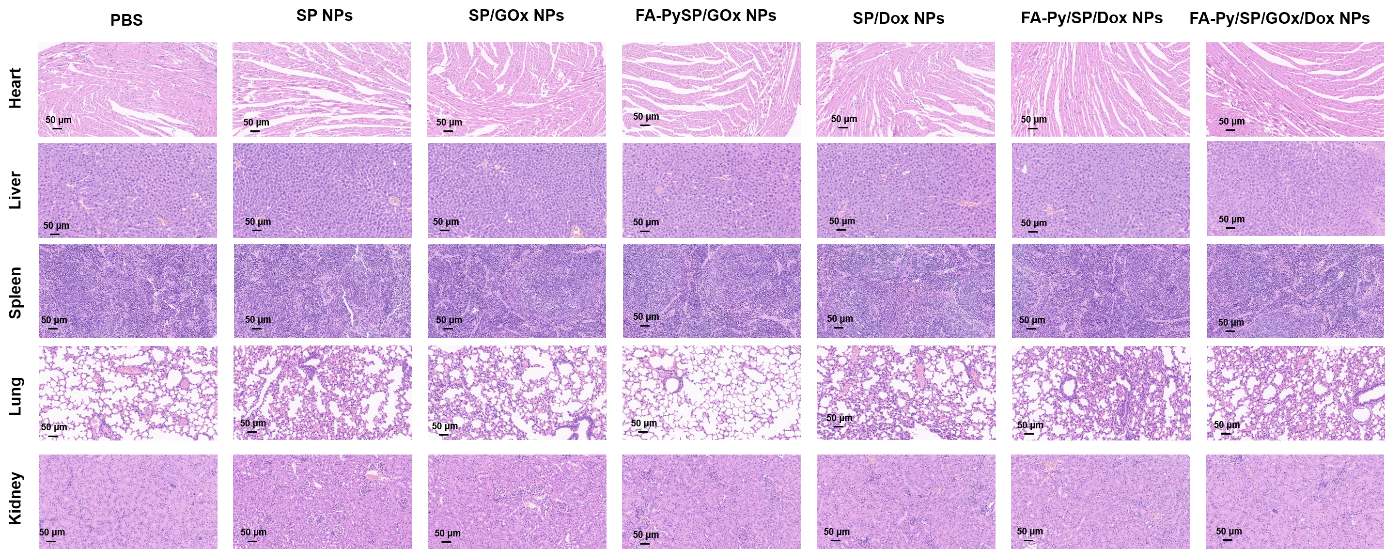


Figure S11. Representative H&E stained images of major organs collected from a untreated mouse (PBS), a SP NPs injected mouse, a SP/GOx NPs injected mouse, a FA-Py/SP/Gox NPs injected mouse, a SP/Dox NPs injected mouse, a FA-Py/SP/Dox NPs injected mouse and a FA-Py/SP/GOx/Dox injected mouse.


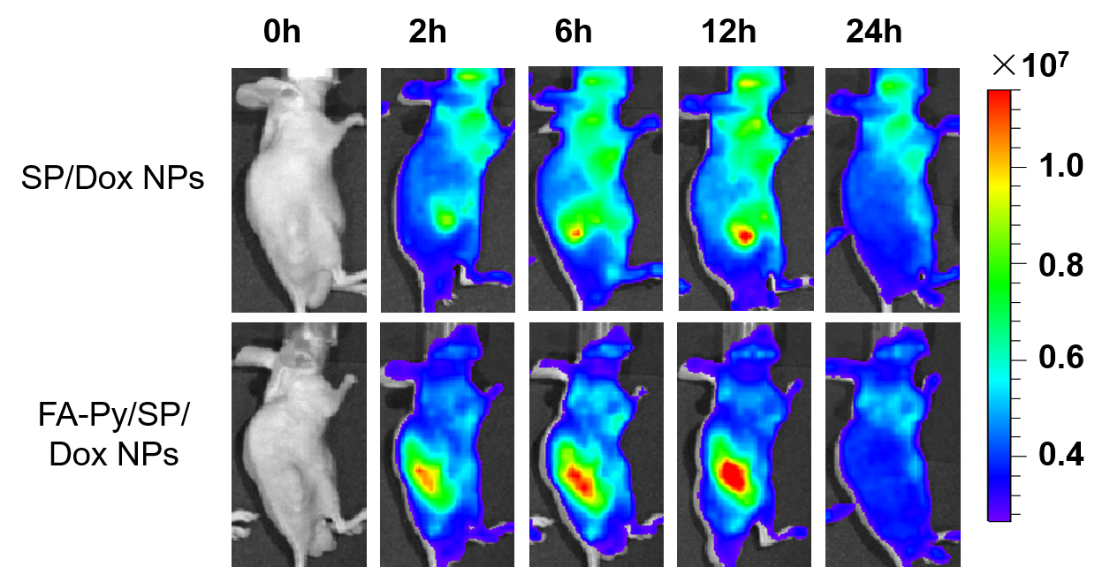


Figure S12. Living imaging of mice after intravenous injection of SP/Dox NPs and FA-Py/SP/Dox at different time periods.


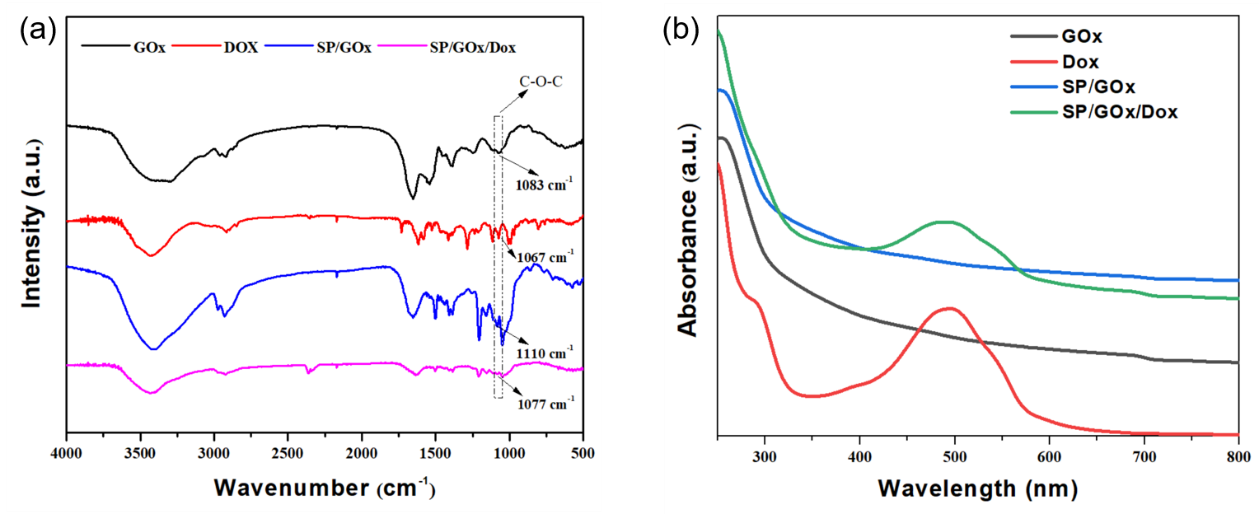


Figure S13. FT-IR (a) and UV (b) spectra of GOx, Dox, SP/GOx and SP/GOx/Dox NPs.
